# Metacognitive Ability and the Precision of Confidence

**DOI:** 10.3389/fnhum.2022.706538

**Published:** 2022-04-13

**Authors:** Keita Somatori, Yoshihiko Kunisato

**Affiliations:** ^1^Department of Psychology, Graduate School of Letters, Senshu University, Kawasaki, Japan; ^2^Department of Psychology, School of Human Sciences, Senshu University, Kawasaki, Japan

**Keywords:** metacognitive ability, metacognitive model, confidence rating, Bayesian cognitive modeling, Bayesian estimation, hierarchical model

## Abstract

In prior research, signal detection theory (SDT) has been widely utilized to assess metacognitive ability. However, the SDT metacognitive model requires the use of a two-alternative forced-choice task, while confidence must also be measured discretely. In our model, participants’ cognitive ability and their confidence in the cognitive task were used to estimate their metacognitive abilities. Therefore, in this study, a metacognitive model that can be applied to various cognitive tasks was developed. This model implements the item response theory (IRT) and *Q*-learning models to estimate cognitive ability; participants’ metacognitive ability is defined as the discrepancy between their confidence in their cognitive ability and their actual cognitive ability. The entire procedure was divided into two experiments. In experiment 1, two different cognitive tasks were used to estimate metacognitive ability and to examine overall discriminative and convergent validity. Notably, the parameters representing metacognitive ability did not correlate with cognitive ability but were positively correlated between the two tasks. In experiment 2, we performed a similar analysis using a different task to test the replicability of experiment 1. The results for experiment 2 were replicated for discriminative and convergent validity, albeit with weak results. Our metacognitive model exhibited high interpretability and versatility.

## Introduction

Metacognition refers to an individual’s perception of their own cognitive processes. Accordingly, Flavell (1979) defined metacognition as the ability to precisely estimate one’s personal cognitive processes. Conversely, Nelson and Narens (1990) argue that metacognition’s main function concerns monitoring and controlling. Specifically, monitoring is a function that oversees and estimates cognitive processes, whereas control is the regulation of any cognitive processes based on gathered information (Koriat and Goldsmith, 1996). Several studies have been conducted based on these definitions of metacognition (Koriat and Goldsmith, 1996; Akturk and Sahin, 2011; Maniscalco and Lau, 2012).

When metacognition is impaired, we lose the ability to accurately perceive our own actions and our environment. As a result, we form false beliefs when faced with uncertainty. Such beliefs act as cognitive biases, leading to faulty reasoning and decision-making. Therefore, metacognition is an important factor in understanding behavioral problems in schizophrenia (Koren et al., 2006; Moritz and Woodward, 2007). Jumping to conclusions and overestimating uncertain events are the main symptoms of schizophrenia, and are problems thought to be caused by impaired metacognition (Koren et al., 2006). Koren et al. (2006) argued that poor metacognition in schizophrenia is associated with other neuropsychological problems (e.g., dysfunction of executive functions or source-monitoring). Rouault et al. (2018) also point out that metacognition is a neuropsychological factor associated with symptoms of various psychiatric disorders, not just schizophrenia. Thus, metacognition is a major outcome for understanding psychiatric disorders from a neuropsychological perspective.

Generally, metacognition has been measured through subjects’ performance in behavioral tasks. For example, Koriat and Goldsmith (1996) implemented a behavioral task comprising three phases: (a) answering a cognitive task, (b) estimating one’s confidence to answer the last question of the particular task, (c) and judging whether to venture a response to the last question. Participants’ scores increased when they answered the last question correctly; conversely, their scores decreased when they answered incorrectly. If a participant opted not to venture a response, their score remained the same. This study revealed that participants should be encouraged to venture a response when they feel more confident.

Moreover, Maniscalco and Lau (2012) suggested that there are two indexes for metacognition, namely, absolute sensitivity and relative sensitivity. Absolute sensitivity is a quantity of evaluated information and accuracy of the metacognitive evaluation. The accurate evaluation of information is nearly synonymous with cognitive performance; that is, if an individual has high cognitive ability, it is easier for them to distinguish when they have erroneously answered a question. Thereby, this situation seems to indicate that the participant has much to share. Additionally, the standard measure of metacognition (the Goodman-Kruskal γ correlation) corresponds to absolute sensitivity (Maniscalco and Lau, 2012), while relative sensitivity only reflects the accuracy of metacognitive evaluation (Maniscalco and Lau, 2012; Fleming and Lau, 2014; Fleming, 2017). Therefore, relative sensitivity does not depend on the type of cognitive tasks or performance.

Notably, relative sensitivity is a useful measure when investigating the relationship between metacognition and other psychological measures (Fleming, 2017). Relative sensitivity enables researchers to differentiate between metacognitive and cognitive ability. For example, Palmer et al. (2014) examined the association between metacognition and aging, and found that cognitive performance decreases over time. Therefore, it is necessary to assess metacognitive ability independently from cognitive ability (Fleming et al., 2010, 2014). Further, Fleming et al. (2014) used relative sensitivity to investigate the neural substrates of metacognition, as it is a suitable index to examine the metacognitive process that distinguishes between cognitive and metacognitive ability.

Maniscalco and Lau (2012) developed the absolute and relative metacognitive index based on the signal detection theory (SDT). In the standard SDT approach, metacognitive ability is defined as the ability to distinguish a correct response from an incorrect one. The SDT approach estimates type-1 *d’* as a cognitive ability on a two-alternative forced-choice (2AFC) task (i.e., a type-1 task). Using this approach, the participants are directed to determine whether or not they have completed the type-1 task and estimate the degree to which they can discriminate between correct and incorrect options in the type-1 task as type-2 *d’*. Moreover, Maniscalco and Lau’s (2012) model estimates meta-*d’* in the type-2 task as well as the type-2 *d*’, as it corresponds to type-2 *d’* in the standard SDT approach. In their approach, relative sensitivity was estimated as meta-*d’*/type-1 *d’* (Maniscalco and Lau, 2012; Fleming and Lau, 2014). They called this measure “metacognitive efficacy” because it expresses the superiority of the type-2 performance, compared with the type-1 performance. Further, Fleming (2017) applied hierarchical Bayesian modeling to the SDT metacognition model to allow its consideration in the context of within and between participants’ uncertainty (e.g., patients vs. controls) and provide a continuous scale for the confidence rating.

Although SDT models are frequently used to evaluate metacognitive ability, they have a few drawbacks. First, the SDT theory assumes that the distribution of signal trial and noise trial has an equal variance; however, this assumption often remains unfulfilled (Fleming, 2017). Second, the SDT task is a 2AFC task, however, it is not used by some metacognitive tasks (e.g., that of Koriat and Goldsmith, 1996). Moreover, a loss of information would occur if it is expressed in terms of a binary scale. The same correct response includes a certain correct answer and chance-level hits, among other aspects. Finally, in the SDT model, confidence has been usually measured through a binary or Likert scale (Fleming et al., 2014; Baird et al., 2015; Fleming, 2017). This dependence is another constraint that the SDT model imposes on the cognitive task. Still, it is possible to measure confidence not only using a binary scale or a Likert scale but also through a continuous range using the visual analog scale (VAS). Continuous scales allow for more accurate measurement of confidence. In summary, the SDT metacognition model imposes the metacognitive task with certain restrictions. Consequently, it is essential to develop a metacognitive model that can be applied to various metacognitive tasks.

Based on our definition of metacognition, we would like to propose a new method to quantify metacognitive ability. Metacognitive ability has been defined as the difference between confidence rating and cognitive performance (Nelson and Narens, 1990; Koriat and Goldsmith, 1996; Georghiades, 2004; Akturk and Sahin, 2011; Maniscalco and Lau, 2012; Yeung and Summerfield, 2012; Fleming and Lau, 2014). That is, the higher the consistency between the subjective assessment of task performance and objective task performance, the greater the evaluated metacognitive ability. Many previous metacognition studies have reaffirmed this definition. Thus, the following question arises: “How do we quantify the discrepancy between objective cognitive performance and subjective confidence?” Further, we divide this question into two sub-questions: “How do we express cognitive performance?” and “How do we express the gap between objective cognitive performance and subjective evaluation?”

In the present study, we attempted to develop a novel metacognitive model that can be applied to various metacognitive tasks, even if they are not 2AFC tasks. Participants can respond to a variety of scales of cognitive performance and confidence. While the SDT model assumes that the variance of confidence for correct and incorrect answers is equal, our model does not require this assumption. We examined the validity of this model from two perspectives. First, we conducted a correlation analysis between the correct ratio and metacognitive ability. As Maniscalco and Lau (2012) posited that relative sensitivity is independent of cognitive performance, the parameter of metacognitive ability should not correlate with the correct ratio. Besides, this analysis is equivalent to discriminant validity, and we conducted a correlation analysis between metacognitive abilities measured through different metacognitive tasks. If metacognitive ability is independent of cognitive performance, it should be measured on the same scale with different tasks. Furthermore, metacognitive ability is a domain-specific function (McCurdy et al., 2013; Fleming et al., 2014; Baird et al., 2015). McCurdy et al. (2013) provided empirical support for these findings by measuring metacognitive ability using perceptual decision and the verbal memory task; they found that the correlation of metacognitive abilities between the two tasks was *r* = 0.47. As metacognitive ability refers to domain-specificity, correlations are lower for different tasks, even if similar concepts are being measured. Therefore, convergent validity is expected to indicate a correlation between the different metacognitive tasks; however, in terms of domain specificity, moderate (rather than high) correlation is expected to appear.

## Materials and Methods

Primarily, two experiments were conducted. In experiment 1, we examined the construct validity of the metacognitive index. Participants engaged in two metacognitive tasks. In experiment 2, we conducted a conceptual replicability test to confirm replication for the results of experiment 1. Additionally, we compared our measure with an existing measure of metacognitive ability developed by Maniscalco and Lau (2012). Before fitting the model to the empirical data in each experiment, simulation and parameter recovery were carried out to examine the behavior of the model parameters and the validity of the estimates. Firstly, we defined cognitive performance as the probability of correct response during trial-by-trial analysis. In our model, cognitive performance was the quantified probability based on the item response theory (IRT). Previous studies have assessed participants’ cognitive performance using a binary scale (Koriat and Goldsmith, 1996; Koren et al., 2006; Fleming et al., 2014; Baird et al., 2015). However, participants’ correct and incorrect responses reflect not only their cognitive abilities but also the difficulty of the task. By applying the IRT model, it is possible to separate participants’ abilities from the factors affecting task difficulty (Baker and Kim, 2004). As a result, it is possible to continuously assess participants’ cognitive performance in each trial. The IRT model estimates participants’ abilities and task difficulty. In each trial, *s*_*th*_ participants’ data were generated as follows (Equation 1):


(1) $$\begin{array}{c} k_{st}∼bernoulli\left(P_{st}\right) \end{array}$$

Here, *P*_*st*_ represents the probability of correct response to the trial *t*_th_. We postulated participant ability (*θ_*s*_*) and item difficulty (*b*_*t*_) to calculate *P*_st_. *P*_*st*_ was calculated as follows (Equation 2):


(2) $$\begin{array}{c} P_{st}∼\frac{1}{1+exp\left(-\left(\theta_{s}-b_{t}\right)\right)} \end{array}$$

This logistic function is often used in the IRT model. As shown in the equation above, the probability of correct response on each trial is denoted by two factors: the participant’s ability and the item’s difficulty. Hence, it is not only applicable to tasks other than the 2AFC but it enables us to consider factors accompanying the task.

In the SDT model, confidence is thought to be distributed with respect to correct and incorrect responses within the cognitive task (i.e., type-2 task). The greater the distance (type-2 *d’*) of the distribution between correct and incorrect trials, the higher the individual’s metacognitive ability (Galvin et al., 2003; Maniscalco and Lau, 2012). In other words, metacognitive ability was considered as the ability to better discriminate between correct and incorrect trials by levels of confidence (Yeung and Summerfield, 2012; Fleming and Lau, 2014). However, participants’ cognitive performance and confidence can also be measured on a continuous scale. For instance, Koren et al. (2006) developed a metacognitive task using the Wisconsin card-sorting test. This task has been used to examine metacognitive ability in patients with schizophrenia (Bruno et al., 2012; Quiles et al., 2015. In Koren et al.’s (2006) task, participants were asked to rate their confidence in the VAS. The SDT model cannot estimate metacognition in a metacognitive task of this type. Consequently, there is a need for a model that can be applied even if the confidence level is continuous, to replace the SDT model.

### Defining the Discrepancy

We defined the discrepancy between objective cognitive performance and subjective evaluation as the variation in confidence in cognitive performance. By thinking of metacognitive ability as a function of reducing the variance of participants’ confidence in the probability of a correct response, we no longer need to assume that there is equal variance with the two distributions. Notably, participants with high metacognitive ability rate their confidence closer to the probability of a correct response, as opposed to participants with low metacognitive ability. Therefore, metacognitive ability indicates the precision of confidence; consequently, a higher metacognitive ability indicates greater precision and confidence closer to the probability of a correct response based on the IRT model.

Bias is a key variable in modeling metacognitive ability. Schraw (2009) classified the indicators obtained by metacognitive judgment into five categories: absolute accuracy, relative accuracy, scatter, discrimination, and bias. All indicators except for scatter quantify the discrepancy between confidence and cognitive performance from various perspectives. The bias index, on the other hand, differs from metacognitive ability in that it relates to the baseline of the confidence rating. Bias represents a certain polarization in the confidence rating, commonly referred to as overconfidence or underconfidence. The higher the bias, the higher the overall confidence rating, and vice versa. Previous research has demonstrated that people make biased judgments in confidence ratings (Ais et al., 2016; Lebreton et al., 2018, 2019). For example, in the study of Lebreton et al. (2018), gain or loss feedback was provided to participants for their responses. In a gain situation, the participants receive money if they answer correctly, but nothing if they answer incorrectly. On the other hand, in a loss situation, participants can avoid losses if they answer correctly, and lose a certain amount of money if they answer incorrectly. Lebreton et al. (2018) showed that people grade their confidence higher in gain than in loss situations. Bias is also taken into account in existing metacognitive models. Fleming and Lau (2014) uses a 2 × 2 matrix to explain the relationship between metacognitive ability and bias. Metacognitive ability is a parameter that discriminates between correct and incorrect trials based on confidence, whereas bias is a parameter that increases or decreases the overall level of confidence. In existing SDT metacognitive models, it has been incorporated into the model as bias.

In light of the existing discussion on bias, we modeled *s*_*th*_ participant’s confidence rating in *t*_th_ trial as Equation 3:


(3) $$\begin{array}{c} confidence_{st}∼Normal\left(100P_{st}+bias_{s},{\frac{1}{meta}}_{s}\right) \end{array}$$

In the above equation, meta*_*s*_* is the metacognitive ability of the *s*th participant, and bias*_*s*_* is the bias of the *s*th participant. To scale the confidence rating, the probability of a correct response (*P*_*st*_) is multiplied by 100. Since confidence is rated from 0 to 100, it is a truncated normal distribution with a lower bound of 0 and an upper bound of 100. That is, we assume that the most rational participant would rate their confidence as the probability of a correct answer multiplied by 100. However, for participants who do not, bias causes their confidence to regularly increase or decrease from the probability of a correct response, with a further constant variation depending on their metacognitive ability. The participants with high metacognition have high precision in their judgment, hence, their standard deviation in confidence distribution is low. Thus, people with high metacognition rate their confidence close to their probability of providing a correct response. Nevertheless, we followed Fleming (2017) to adopt a hierarchical model approach for estimating metacognitive ability as Equation 4:


(4) $$\begin{array}{c} meta_{s}∼Normal\left(\mu_{meta},\sigma_{meta}\right) \end{array}$$

In this way, the metacognitive ability of each participant can be estimated more reliably than if they are estimated independently. In Equation 4, *μ*_meta_ and *σ*_meta_ indicate group-level parameters for metacognitive ability. We assumed that each participant’s metacognitive abilities were normally distributed around *μ*_meta_.

A difference between the model of Maniscalco and Lau (2012) and our model is the methodology used to measure cognitive performance and the discrepancy between performance and confidence. To measure cognitive performance, our model used the probability of a correct response as estimated by the IRT model, whereas Maniscalco and Lau’s (2012) model used correct and incorrect responses. To measure the discrepancy between performance and confidence, our model used precision in confidence rating, whereas Maniscalco and Lau’s (2012) model used meta-*d’*/type-1 *d*’. The IRT model solved the problem of cognitive performance being aggregated into 0 or 1 by using cognitive performance. We used the probability of a correct response estimated in each trial, rather than the behavioral data of correct and incorrect responses. The probability of a correct response provides the reference point for the participant’s confidence rating. Therefore, we can treat cognitive performance continuously without restrictions, such as the homogeneity of variance for noise and signal distribution. Our model also renders metacognitive ability easier to interpret compared with the SDT model. In our model, the higher the metacognition, the more accurate the confidence rating for the probability of a correct response. Finally, we interpret metacognitive ability as the accuracy of confidence ratings for the probability of a correct response.

### Experiment 1

The purpose of study 1 was to affirm the validity of the metacognitive model. We selected general knowledge questions and visual memory exercises for metacognitive tasks. The metacognitive general knowledge task was prevalent in the metacognitive study (Koriat and Goldsmith, 1996; Danion et al., 2001), followed by the metacognitive memory task.

#### Participants

Seventy-eight undergraduates and graduate students [35 men, 43 women; mean age = 21.13 years (SD = 1.65)] participated in this study. All candidates provided their written informed consent before participating in this study. The study design was approved by the Ethics Committee on Research with Human Participants at Senshu University (14-ML147004-1).

#### Metacognitive General Knowledge Task

**FIGURE 1:**
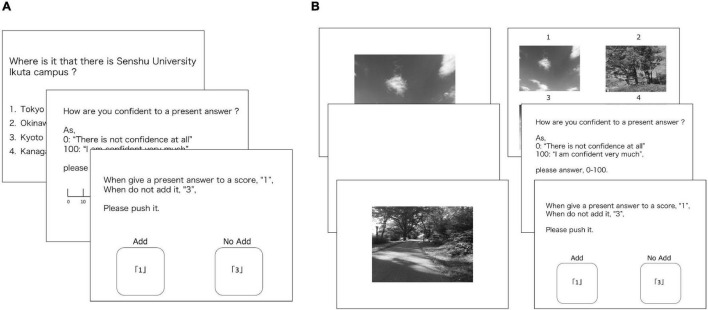
**(A)** Metacognitive general knowledge task. **(B)** Metacognitive recognition task.

A metacognitive task was developed to enable participants’ (a) response to general knowledge questions, (b) estimation of confidence in the last answer, and (c) judgment on whether they should venture a response to the last question (Koriat and Goldsmith, 1996; Figure 1A). General knowledge questions relied on a collection of questions related to an employment examination. As the collection we referred to encompassed five items, we excluded one option according to the metacognitive task of Koriat and Goldsmith (1996). Further, the general knowledge questions were presented in a random order. Confidence estimation was also conducted ranging from 0 (“not confident at all”) to 100 (“very confident”). Participants were informed that if they answered the last question correctly, their total score would increase, but if their response attempt was incorrect, their total score would decrease, and that if they did not venture a response, their total score would remain unchanged. Additionally, participants were instructed to obtain the highest score possible. We did not use the data from the venturing phase in the metacognitive model proposed in this study. Moreover, to control the feedback effect, the participants were not presented with their total score.

We presented the stimuli on a PC monitor, while participants pressed buttons and used a computer mouse to register their responses. Participants had to press a button numbered 1 to 4 in order to answer a general knowledge question. They also used the mouse to indicate their confidence rating and venture a response to the items in the test. All phases were carried out at the participant’s own pace and took approximately 10–20 m to complete, depending on the individual.

#### Metacognitive Visual Recognition Task

The metacognition tasks we conducted used recognition-related questions including memorization and recognition (Figure 1B). For encoding, sixty pictures were presented in random order. Each picture remained onscreen for 500ms, while the inter-trial-interval lasted 3,000 ms. The recognition phase also entailed participants’ (a) response to recognition questions, (b) estimation of confidence in the last answer, and (c) judgment on whether they should venture a response to the last question. Moreover, recognition-related questions comprised four options that targeted stimuli-presented memorization and three distractors. We selected the picture stimuli from the International Affective Pictures System (IAPS) (Lang et al., 2008), and selected three emotional valences (positive, neutral, and negative) per 80 pieces, based on the framework of Liu et al. (2008). As with the metacognitive general knowledge task, we presented the stimuli on a PC monitor and used buttons and a mouse to register participants’ responses. All phases were conducted at the participant’s own pace and took between 5 and 15 min.

#### Procedure

After submitting their informed consent, participants engaged in two metacognitive tasks and completed several questionnaires^1^. The task order was counterbalanced (see Endnote 1), and all participants were randomized. Prior to starting the experiment, participants practiced the first metacognition task that they would be tested in.

#### Simulation

We conducted a simulation of our metacognitive model behavior. We set the probability of obtaining a correct answer from 0 to 1, which was assumed to be the same for all participants, while metacognitive ability were set to 0.01 or 0.1 and bias were set to −20 or 20 to generate the simulation data. We retrieved the simulation data of four participants with divergent metacognitive ability. Thereafter, our models conducted an 80 trial cognitive task with varying degrees of difficulty and rated participants’ confidence in the last answer on each trial. Note that participants’ cognitive abilities (i.e., Equation 2) were assumed to be identical. As a result of our simulations, we visualized the probability of a correct response and confidence for the four simulated participants.

#### Parameter Recovery

We checked a parameter recovery on our model, which evaluates the goodness for estimating the method of applying the cognitive model (Busemeyer and Diederich, 2010). First, we set the true value of each parameter as follows: *μ*_meta_ = 0.1, *σ*_meta_ = 1, *θ_s_* ∼ uniform (0, 5), *b*_*t*_ ∼ normal (0, 5), bias_s_ ∼ normal (0, 5). From the values of these parameters, we generated data for 100 participants according to our model. We used this data to estimate the metacognitive abilities of each participant. The goodness of the parameter recovery was examined via correlation analysis between the true and estimated values of the metacognitive ability parameters. The details of parameter estimation are presented below.

#### Data Analysis

We used the data analysis software R3.6.0 (R Core Team, 2019). The estimation for the parameter was conducted through Bayesian estimation. The Hamiltonian Monte Carlo Algorithm was adopted using the rstan package (version 2.18.2; Stan Development Team, 2018). The Markov chain Monte Carlo (MCMC) sample size was 20,000 (iteration = 10,000, warm-up = 5,000, thin = 1, and chain = 4). The convergence diagnosis was based on Rhat statistics (Gelman and Rubin, 1992). We adopted the above setting for all parameter estimates in this study. Afterward, we conducted a correlation analysis between the correct ratio and metacognitive parameter as discriminant validity, and correlation analysis between the two metacognitive tasks as convergent validity. We will also examine the correlation between the estimated metacognitive abilities and biases. Additionally, we used BayesFactor packages (version 0.9.12-4.2) (Morey and Rouder, 2018) to estimate the correlation coefficient and Bayes factor. In all correlation analyses, the Bayes factor was compared between models with *r* = 0 and models with *r* ≠ 0.

### Experiment 2

In experiment 2, to gauge conceptual replicability, we developed a meta verbal memory task and a novel meta reversal learning task. Memory tasks are often used to estimate metacognition (McCurdy et al., 2013; Fleming et al., 2014; Baird et al., 2015). For the verbal memory task, we developed the Japanese version of a verbal memory task based on McCurdy et al. (2013). We also developed an original metacognitive task using a reversal learning task. Although we are interested in measuring metacognition in the learning process, we could not find the meta-learning task. Hence, we need to measure metacognitive ability under the given circumstances. Further, a simple learning task would be too easy for participants and could inflate their confidence level. Therefore, we used a reversal learning task to ensure that the confidence level was wide-ranging. In Study 2, we conducted a total of four analyses. Two of them were correlational analyses of the estimated metacognitive ability in each cognitive task, with the performance in the cognitive tasks and correlational analyses of the metacognitive abilities across cognitive tasks, in order to gauge the conceptual replicability of Study 1. The other two analyses were comparisons with existing estimation methods. The cognitive tasks used in Study 2 are both 2AFC tasks. Therefore, the method using the SDT, proposed by Maniscalco and Lau (2012), can be applied therein. Thus, we carried out the same correlation analysis with participants’ performance in each task as well as between tasks, based on the metacognitive ability estimated by the SDT model. The comparison between the first two analyses and the latter two allows us to verify the superiority of the proposed model over the existing methods.

#### Participants

Sixty-six undergraduates and graduate students [male = 25, female = 41; mean age = 19.79 (SD = 1.00)] took part in this study. All participants provided written informed consent before the experiment. This study was approved by the Ethics Committee on Research with Human Participants at Senshu University (16-DL167002-1).

#### Metacognitive Verbal Recognition Task

**FIGURE 2:**
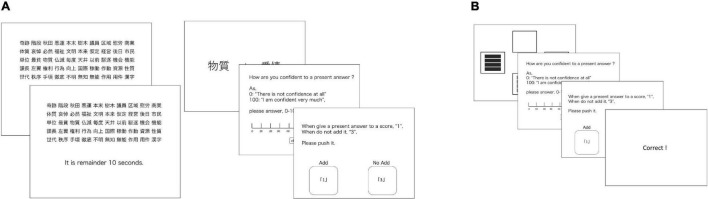
**(A)** Metacognitive reversal learning task. **(B)** Metacognitive verbal memory task.

Metacognition exercises that depend on memory tasks included encoding and recognition (McCurdy et al., 2013; Figure 2A). This task consisted of four blocks. In the initial blocks, the participants were shown 50 words. We used Japanese words from the NTT database (Amano and Kondo, 2008). This memorization phase lasted 30–60 s (the duration of which was randomly assigned for each block). After memorization, three phases of the metacognitive task followed: (a) answering the recognition questions, (b) estimating the confidence in the last answer, and (c) judging whether to venture a response to the last question. The recognition questions followed the 2AFC format. The participants were introduced to both a target and a distractor word and were asked for a target word of their choice that had been presented during the memorization phase. Each block consisted of 20 trials, yielding a total of 80 trials. As with the metacognitive general knowledge task, we presented the stimuli on a PC monitor and used buttons and a mouse to register participants’ responses. All phases were conducted at the participant’s own pace and took approximately 20 min.

#### Metacognitive Reversal Learning Task

The metacognition task that utilized the reversal learning procedure consisted of 3 phases (Figure 2B) and was based on Chamberlain et al. (2006). In the reversal learning task, participants were asked to choose one of two options and to learn which stimuli were associated with reward. First, two stimuli were displayed in two of four randomly selected locations. Subsequently, the participants were asked to select a single option, and to conclude the trial; the participants received feedback regarding whether their answers were correct or incorrect. Furthermore, during the acquisition process, the correct option resulted in a 70:30 ratio of reward/punishment. Conversely, the incorrect option resulted in a 30:70 ratio of punishment/reward. After 40 trials, the reinforcement contingency was reversed. In the flow of the task, participants were asked to (a) choose one of two options located in four randomly selected locations, (b) estimate their confidence in the last answer, and (c) judge whether they should venture a response to the last question. Notably, (b) and (c) were parts of the same procedure in the metacognitive memory task, while the participants received probabilistic feedback at the end of each trial. The procedure for this experiment was similar to the previous one.

#### Procedure

After participants submitted their informed consent, they engaged in two metacognitive tasks. Before the task, participants had to answer several questionnaires^2^. Subsequently, the task order was counterbalanced, and all participants were assigned randomly. Additionally, a practice trial was conducted before each task.

#### Simulation

We simulated our metacognitive model behavior again. Since no trials were independent in the reversal learning task, it is not appropriate to use the IRT model to figure the probability of the correct choice. Therefore, instead of the probability of correct response, we used the *Q* value estimated by the *Q*-learning model as a measure of cognitive performance (Katahira, 2015). In the *Q*-learning model, the *Q* value is updated by Equation 5:


(5) $$\begin{array}{c} Q_{st}=Q_{st-1}+\alpha_{s}^{L}\left(feedback_{st}-Q_{st-1}\right) \end{array}$$

In this equation, the subscript *s* stands for participant *t* in the trial. Participants choose one of two alternate opportunities in each trial and receive feedback. The participants have a *Q* value for each choice, and the *Q* value is updated based on the feedback. The magnitude at which the updated *Q* value is stipulated is through the learning rate *α*^L^** of the participants. By contrast, the *Q* values of the occurrence when participants did not choose any option decayed according to the participant’s forgetfulness rate (*α*^F^**). Next, the probability of participants choosing each option is calculated by using the *Q* value as follows Equation 6:


(6) $$\begin{array}{c} choiceprobability_{st}=\frac{1}{1+exp\left(\beta_{s}\left(Q_{st}^{option1}-Q_{st}^{option2}\right)\right)} \end{array}$$

Here, *β_*s*_* represents the randomness of the participants; the higher the value of *β_*s*_*, the greater its sensitivity in terms of the choice probability between the two possible options. Thus, the choice probability reflects the participant’s learning abilities and inconsistencies.

Following the balance theory, Lebreton et al. (2019) modeled the confidence in a learning task as follows. The balance theory assumes that evidence for a given option as being accurate accumulates independently for each option, and that a decision is made when a certain threshold is crossed (Vickers, 2001). At this point, the degree of confidence is the difference in the evidence for each option at the time the decision is made. In other words, the greater the difference between the correctness of the two alternatives, the higher the confidence level. One view that differs from the balance theory is that the degree of confidence in each option is evaluated independently. Zylberberg et al. (2012) showed that in a perceptual task, confidence in one option is not affected by evidence for the other option. This can be said to be a refutation of the balance theory. However, the task we adopt in this study is a forced two-choice learning task, which means that one of the choices is always the correct answer. Therefore, it is natural to assume that the degree of confidence in one option is affected by the correctness of the other option. Therefore, in this study, we represent the process of confidence based on the balance theory.

On the other hand, there is a modification to be made in introducing Lebreton et al. (2019) into our study. Lebreton et al. (2019) incorporates an intercept parameter in order to express confidence in a regression model. However, since we would be modeling metacognitive ability, we still need to incorporate the bias parameter, which is the overall level of confidence. When the intercept parameter and the bias parameter coexist, there is a concern that the parameters will be less discriminative and will not converge. Therefore, in our study, we treat the intercept term as a bias parameter and formulate it as follows.


(7) $$\begin{array}{c} confidence_{st}∼Normal(b_{1}\Delta Q_{st}\\ \;+b_{1}confidence_{st-1}+bias_{s},\frac{1}{meta_{s}}) \end{array}$$

In Equation 7, Δ*Q* is the absolute value of the difference between the *Q* values of the two alternatives. Δ*Q* is expressed as follows:


(8) $$\begin{array}{c} \Delta Q_{st}=abs\left(Q_{st}^{option1}-Q_{st}^{option2}\right) \end{array}$$

Equations 7 and 8 express that the confidence level is obtained by a regression model that includes the difference in evidence between the alternatives based on the balanced model, the confidence level before one trial, and the bias. Further, metacognitive ability, like the model in experiment 1, is the precision for the value that the confidence should take. Additionally, we created a hierarchical model for metacognitive ability according to Equation 9:


(9) $$\begin{array}{c} meta_{s}∼Normal\left(\mu_{meta},\sigma_{meta}\right) \end{array}$$

Using the *Q*-learning model presents another advantage that allows us to test the generality of our metacognitive model. If the IRT model is the only way to estimate participants’ cognitive performance, the cognitive task used to estimate metacognitive ability is limited. However, if the metacognitive ability can be estimated in the same way using the *Q*-learning model, it can be applied to a variety of functions, including learning tasks.

Since we used a different model in experiment 1, we again simulated the behavior of the model. The values for each parameter were set in advance and the simulation data was created. We assumed a situation in which four participants with different metacognitive abilities and biases would participate in a learning task consisting of 80 trials. The relationship between each participant’s confidence level, metacognitive ability, and bias was visualized.

#### Parameter Recovery

We checked a parameter recovery on our model and set the true value of each parameter as follows: *μ*_meta_ = 0.1, *σ*_meta_ = 1, bias ∼ normal (50, 10), *α*^L^_s_ ∼ beta (2, 1), *α*^F^_s_ ∼ beta (2, 1), *β_*s*_* ∼ uniform (0, 10), *b*_1_ = 20, *b*_2_ = 0.1. From the values of these parameters, we generated data for 100 participants according to our model. Thereafter, we used this data to estimate the metacognitive ability of each participant. The goodness of the parameter recovery was examined by correlation analysis between the true and estimated values of the metacognitive ability parameters. The details on parameter estimation are presented below.

#### Data Analysis

In total, our study comprised 58 participants for the following analysis. As eight participants were correct at a rate of less than 0.5 (i.e., level in a chance), we determined that the participants were not very keen about the task. We used a data analysis environment of the R3.6.0 program (R Core Team, 2019). The estimation for the parameter was conducted through the Bayesian estimation. Besides, the Hamiltonian Monte Carlo Algorithm was adopted using the rstan package (version 2.18.2) (Stan Development Team, 2018). The MCMC sample size was 20,000 (iteration = 10,000, warm-up = 5,000, thin = 1, and chain = 4) and the convergence diagnosis was based on Rhat statistics (Gelman and Rubin, 1992), and was validated. To estimate the metacognitive ability based on the SDT model, we used the script developed by Fleming, which allows Bayesian estimation of meta-*d’* using JAGS^1^. JAGS is a library for Bayesian estimation using MCMC sampling (Plummer, 2003). While Fleming’s script assumes a cognitive task in which both signal and noise trials are performed, the present study employs the cognitive tasks in which only signal trials are performed. Therefore, we made some changes to the script with reference to Mazancieux et al. (2020). The MCMC sample size was the same as in the case of rstan. The Rhat statistic was used for convergence diagnosis as in the proposed model. The metacognitive ability was obtained by dividing the estimated meta*-d*’ by *d*’. Experiment 2 was carried out to examine reproductivity; hence, an analysis similar to experiment 1 was conducted. We also used BayesFactor packages (version 0.9.12-4.2) (Morey and Rouder, 2018) to estimate the correlation coefficient and Bayes factor.

## Results

### Experiment 1

#### Simulation and Parameter Recovery of the Metacognitive Model

**FIGURE 3:**
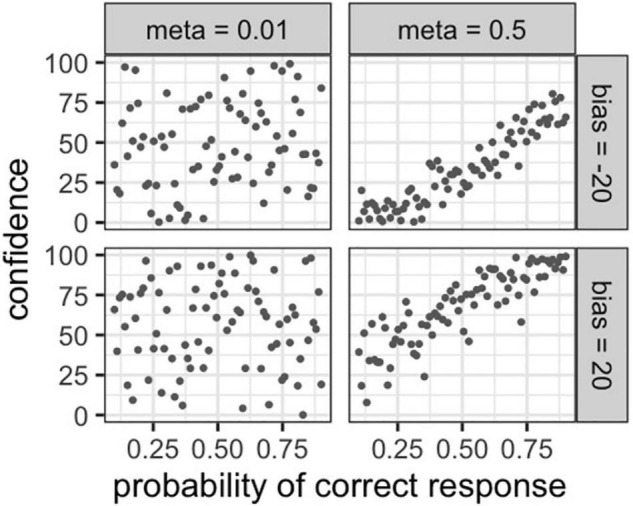
Simulation of the association between the probability of a correct answer and confidence in different metacognitive ability and bias.

Figure 3 depicts the performance of four participants with different metacognitive abilities on the same cognitive task. Their probability of correct response and confidence for each trial were simulated. High-metacognition participants (meta = 0.1) rated their confidence near their respective probability of a correct response. Accordingly, low-metacognition (meta = 0.01) participants rated their confidence without relation to their probability of a correct response. In addition, participants with high bias (bias = 20) rated their confidence higher overall, while participants with low bias (bias = −20) rated their confidence lower.

**FIGURE 4:**
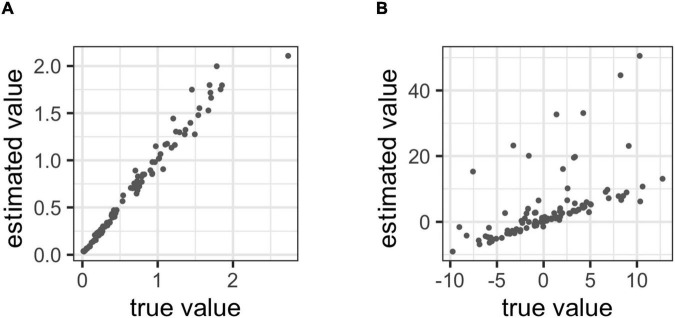
**(A)** Correlations between the true values and estimated value in metacognitive ability, **(B)** Correlations between the true values and estimated value in bias.

Figures 4A,B shows that the true parameter value we set and the parameter values estimated by Bayesian estimation were moderately to strongly correlated (meta: *r* = 0.985; bias = 0.583). Therefore, this suggests that the parameters of the model can be adequately projected using Bayesian estimation.

#### Discriminatory and Concomitant Validity of Metacognitive Ability

**FIGURE 5:**
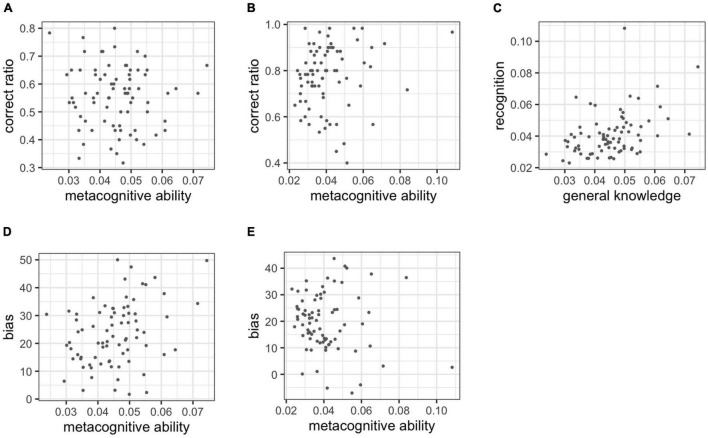
**(A)** Correlations between the correct ratio and metacognitive ability in the general knowledge task, **(B)** correlation between the correct ratio and metacognitive ability in the recognition task, **(C)** correlation with metacognitive ability between tasks, **(D)** correlations between metacognitive ability and bias in the general knowledge task, **(E)** correlations between metacognitive ability and bias in the recognition task.

The correlation analysis with the metacognitive abilities was estimated and the correct ratio was provided for Figures 5A,B. However, metacognitive ability did not correlate with cognitive performance in each task. Moreover, in the general knowledge task, *r* = −0.060 [95% confidence interval (CI): −0.277 to 0.154; Bayes factor (BF) = 0.303]. In the recognition task, *r* = 0.148 (95%CI: −0.064 to 0.361; BF = 0.654). The correlation analysis with metacognitive ability in the two tasks is shown in Figure 5C. A medium correlation was observed between the metacognitive ability of each task, *r* = 0.399 (95%CI: 0.200 to 0.600; BF = 208.587).

Figures 5D,E shows the correlation between metacognitive ability and bias. The correlation was observed in the general knowledge task, *r* = 0.305 (95% CI: 0.092 to 0.507; BF = 13.253). In the recognition task, *r* = −0.108 (95%CI: −0.321 to 0.107; BF = 0.416).

### Experiment 2

#### Simulation and Parameter Recovery of the Metacognitive Model

**FIGURE 6:**
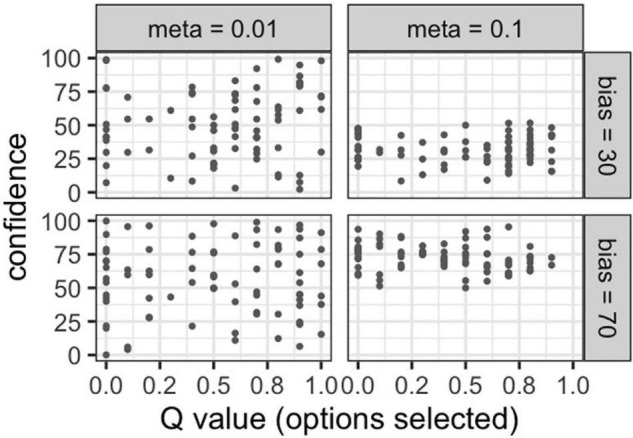
Simulation of the association between the probability of a correct answer and confidence in different metacognitive abilities.

As in experiment 1, we plotted the possibility of a correct response and the confidence of the five participants with different metacognitive abilities in Figure 6. Participants with higher metacognitive ability rated their confidence closer to their respective probability of a correct response. In addition, participants with high bias (bias = 70) rated their confidence higher overall, while participants with low bias (bias = 30) rated their confidence lower.

**FIGURE 7:**
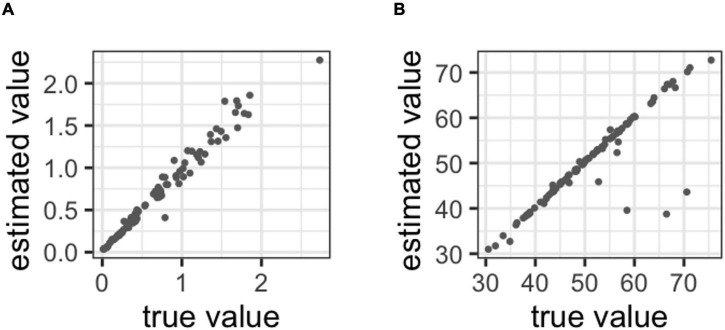
**(A)** Correlations between the true values and estimated value in metacognitive ability, **(B)** correlations between the true values and estimated value in bias.

Similarly, Figures 7A,B shows that the true parameter value we set and the parameter value estimated by Bayesian estimation were strongly correlated (meta: *r* = 0.986; bias = 0.890). Therefore, this indicates that the parameters of the model can be adequately estimated using Bayesian estimation.

#### Discriminatory and Concomitant Validity of Metacognitive Ability

**FIGURE 8:**
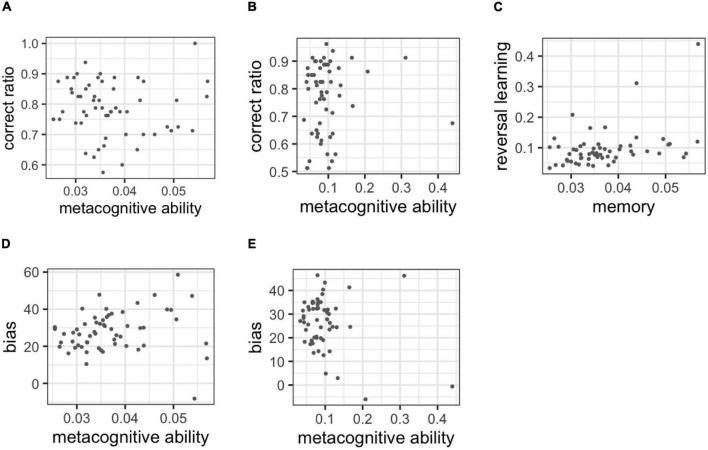
**(A)** Correlations between the correct ratio and metacognitive ability in the memory task, **(B)** correlation between the correct ratio and metacognitive ability in the reversal learning task, **(C)** correlation with metacognitive ability between tasks, **(D)** correlations between metacognitive ability and bias in the memory task, **(E)** correlations between metacognitive ability and bias in the reversal learning task.

The correlation analysis with the metacognitive ability was estimated and the correct ratio was also provided for Figures 8A,B. Metacognitive ability did not correlate with cognitive performance in each task. In the memory task, *r* = −0.030 (95%CI: −0.269 to 0.217; BF = 0.306) and in the reversal learning task, *r* = 0.063 (95%CI: −0.182 to 0.312; BF = 0.340). The results of experiment 1 on discriminant validity were replicated.

The correlation analysis with the metacognitive ability in the two tasks is also presented in Figure 8C. Lastly, the metacognitive ability indicated a medium correlation between tasks [i.e., *r* = 0.368 (95%CI: 0.136 to 0.592; BF = 21.115)].

Figures 8D,E shows the correlation between metacognitive ability and bias. The correlation was observed in the memory task, *r* = 0.133 (95% CI: −0.113 to 0.374; BF = 0.534). In the reversal learning task, *r* = −0.226 (95%CI: −0.462 to 0.020; BF = 1.565).

**FIGURE 9:**
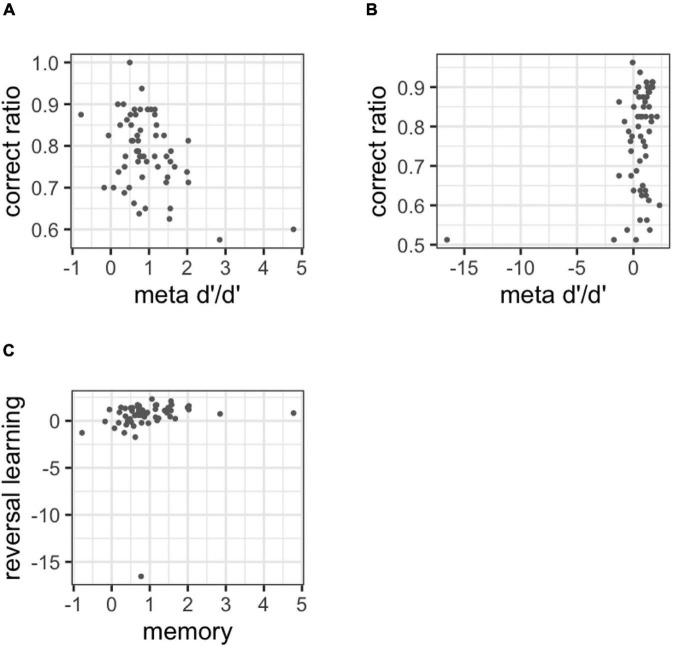
**(A)** Correlations between the correct ratio and meta-*d*’/*d*’ in the memory task, **(B)** correlation between the correct ratio and meta-*d*’/*d*’ in the reversal learning task, **(C)** correlation with meta-*d*’/*d*’ between tasks.

Figure 9 shows the results of the discriminant and convergent validity for meta-*d*’/*d*’. As evident in Figures 9A,B, meta-*d*’/*d*’ was found to be correlated with the correct ratio, *r* = −0.400 (95%CI: −0.631 to −0.152; BF = 54.598), *r* = 0.300 (95%CI: 0.056 to 0.536; BF = 5.263). In addition, the correlation between tasks (Figure 9C) was positive, but the 95% confidence interval included 0, *r* = 0.130 (95%CI: −0.112 to 0.364; BF = 0.505).

## Discussion

Typically, a model based on the SDT is used to measure metacognitive ability (Maniscalco and Lau, 2012; McCurdy et al., 2013; Fleming and Lau, 2014; Baird et al., 2015); however, the SDT model is suitable for tasks that measure cognitive performance in a 2AFC task and confidence in the discrete scale. Further, some metacognitive tasks do not meet the assumptions and constraints of the SDT metacognitive model (Koriat and Goldsmith, 1996; Koren et al., 2006). Therefore, in the present study, we established a new metacognitive model that does not depend on similar constraints as the SDT model. In our model, participants’ cognitive abilities were derived using the IRT and *Q*-learning model; we estimated the metacognitive ability as the precision of cognitive performance. Further, our model enables the assessment of participants’ responses and confidence with various measures.

We systematized a metacognitive model based on examining discriminant validity and construct validity. Maniscalco and Lau (2012) argued that there are two aspects of metacognition, namely, absolute sensitivity and relative sensitivity. Absolute sensitivity might serve as a measure of metacognition; however, the measure of relative sensitivity has not been comprehensively investigated. As such, we conducted modeling to that process based on the theoretical framework of metacognition (Flavell, 1979; Koriat and Goldsmith, 1996; Koren et al., 2006; Fleming and Lau, 2014).

In experiment 1, our metacognitive measure did not correlate with the correct ratio. Therefore, our metacognitive measure was independent of cognitive performance. This finding specified that our parameter of metacognitive ability has discriminant validity. Contrariwise, the previous study contended that relative metacognition has to be independent of cognitive ability (Maniscalco and Lau, 2012), as we do not know what the measure imitates if the measure correlated with the correct ratio. In addition, the correlation between metacognitive measures and the correct ratio contradicted the definition of relative metacognition (Maniscalco and Lau, 2012). Nevertheless, experiment 2’s results replicated the results of experiment 1. Among different participants and different tasks, the discriminant validity of our model was fulfilled. Particularly, the applicability of our metacognitive model was demonstrated even for a task that was not undertaken.

Lastly, our metacognitive model fulfilled the criterion of Maniscalco and Lau (2012) from the perspective of discriminant validity. In our model, metacognitive ability did not correlate with cognitive performance in four different tasks, in line with prior research on metacognition (Nelson and Narens, 1990; Koriat and Goldsmith, 1996; Georghiades, 2004; Akturk and Sahin, 2011; Maniscalco and Lau, 2012; Yeung and Summerfield, 2012; Fleming and Lau, 2014). That is, metacognitive ability is the divergence between objective cognitive performance and subjective evaluation (e.g., confidence estimation). Moreover, in our model, the probability of a correct response or *Q* value indicates participants’ estimated cognitive performance, while confidence is obtained from the distribution centered on these cognitive performances. The precision of confidence distribution is assumed as the metacognitive ability. Accordingly, metacognitive ability has a high precision when the variance of the distribution is small. As individuals’ confidence is close to their estimated performance, it can be said that objective performance can be accurately evaluated. Lastly, the parameter recovery and simulation reflect these processes; hence, our model is considered to be an accurate model of metacognition.

Another advantage of our model is its high interpretability. Prior researchers have argued that the cognitive model should be simpler and more interpretable (Wilson and Collins, 2019). In the model illustrated by Maniscalco and Lau (2012), the metacognitive ability was calculated as meta-*d*’/type-1 *d*’. Therefore, independence is ensured by dividing absolute metacognitive ability by objective performance. However, this operation is difficult to interpret as a cognitive parameter. Thus, our model was designed so that metacognitive ability is expressed more directly. We simply consider that the higher the metacognitive ability, the more congruent the cognitive ability and confidence ratings. As such parameters are defined directly in our model, it is also easy to interpret that the degree of metacognitive ability influences the variation of confidence rating.

The superiority of our model over the standard SDT model is not only shown by its ease of interpretation but also by empirical data. The experimental task used in experiment 2 was a 2AFC task to which the SDT model was applicable. Therefore, we conducted the same analysis for the SDT model in experiment 2. The results showed that the metacognitive ability estimated by the SDT model (meta-*d*’/*d*’) was correlated with the correct ratio. This means that the definition of relative sensitivity was not met and that the SDT model did not provide appropriate estimates for metacognitive ability. Further, the correlations between tasks were not sufficiently high, while the stability of the estimates was threatened.

It may be difficult to apply the SDT model to confidence data measured using a VAS. The relative sensitivity estimated in the SDT model was developed to quantify metacognitive ability, which is not correlated with objective cognitive performance, but was not characterized in this study. This may be due to the fact that the range of confidence ratings was too fine. As documented in https://github.com/metacoglab/HMeta-d, a matrix of confidence × stimulus × response was created as data for the estimation of meta-*d*’ (Fleming, 2017). Referring to the example given in Fleming (2017), if the confidence ratings comprise three levels, nR_S1 = (100, 50, 20, 10, 5, 1). In this case, the first group (100, 50, 20) represents the confidence counts for response A to stimulus A, while the second group (10, 5, 1) represents the confidence counts for response B to stimulus A. For one participant, we created such vectors for each stimulus, A and B, and included them as data. In this study, confidence is measured using a VAS from 0 to 100, which means that it is measured with a 101 level of confidence rating. Therefore, creating vectors as described above will generate a very large number of 0 cell counts. This may have made the estimation of the meta-*d*’ difficult and, consequently, unsuccessful. Mazancieux et al. (2020) asked the participants to rate their confidence on a scale of 0 to 100, divided into sections of 10 (e.g., 0–10 and 11–20). This suggests that it may be difficult to apply the SDT model if there are too many cells for confidence ratings.

Our metacognitive measure also satisfies convergent validity. In experiment 1, the correlation with both tasks indicates that our metacognitive model reflects similar concepts. Subsequently, our measure represents metacognition independently of the task selected. These results are replicated in experiment 2, which exhibited a positive correlation between tasks. On the other hand, these indicators were only estimated for different tasks and should reflect the same capabilities. Nevertheless, the correlation coefficient is moderate and not high. This could be because metacognitive ability is domain-specific (McCurdy et al., 2013; Baird et al., 2015). Furthermore, in McCurdy et al. (2013), the correlation between metacognitive ability in perceptual and memory tasks was *r* = 0.47. Given that we are measuring the same concept, this correlation was not high. The same study in Baird et al. (2015) was also examined, but the correlation coefficient was low (*r* = 0.15). This is presumably due to domain specificity.

In recent years, many studies have researched the relationship between metacognitive ability and other psychological measures (Koren et al., 2004; Bruno et al., 2012; Fleming et al., 2014). In these cases, it is necessary to measure only metacognitive ability. This measure may not correlate with cognitive performance; however, our metacognitive model meets this criterion. For example, the contribution of metacognitive ability might be more clearly seen by considering the study by Koren et al. (2006), who examined the association between mental illness and metacognitive ability. Moreover, in this research context, it would be imperative to measure metacognitive ability with a more interpretable measure. In our model, metacognitive ability refers to the precision of confidence in cognitive performance. In other words, high metacognitive ability means that confidence levels more accurately capture cognitive performance. Hence, such ease of interpretation is another benefit of our model.

In existing frameworks for metacognitive measures, we can also situate the metacognitive abilities that we estimate in our model. For example, Maniscalco and Lau (2012) distinguish between absolute and relative sensitivity for metacognitive measures. In this regard, as mentioned in the introduction, our model corresponds to relative sensitivity. On the other hand, in the framework of Schraw (2009), it is considered to correspond to an index called absolute accuracy. Schraw (2009) classified metacognitive measures into five categories: absolute accuracy, relative accuracy, bias, scatter, and discrimination. In this framework, absolute accuracy is represented by the absolute difference between cognitive performance and confidence. It is similar to our model in that it directly deals with the discrepancy between cognitive performance and confidence. Relative accuracy, on the other hand, is represented by the correlation between cognitive performance and confidence. Therefore, it is stated that these two indices do not necessarily coincide, and each reflects a different aspect of metacognitive ability. Although relative sensitivity can be said to represent high metacognitive ability, it differs from our model because it does not necessarily deal directly with the discrepancy between cognitive performance and confidence. Bias is determined by an equation roughly analogous to that of absolute accuracy, representing whether the average level of confidence is high (overconfidence) or low (underconfidence) relative to performance. On the other hand, modeling with Schraw (2009) does not clearly distinguish between metacognitive ability and bias. Scatter is not necessarily a measure of metacognitive ability; it is expressed as the difference between the variance of confidence in correct and incorrect trials. In other words, it is positive if the variance of confidence in the correct answer trials is relatively large, and negative if it is small. It can be regarded an indicator of metacognitive judgment in the sense that it expresses one aspect of confidence, but it does not express the level of metacognitive ability. Thus, our model is a metacognitive model based on the discrepancy between subjective evaluation and cognitive performance as expressed by absolute accuracy.

In a metacognitive general knowledge task, positive correlations with metacognitive ability and bias were shown. In Fleming and Lau (2014), it appears that metacognitive ability and bias are assumed to have independent effects on confidence. Some studies suggest that metacognitive ability and bias independently affect confidence (e.g. Boldt et al., 2017), but they do not examine direct correlations and are therefore insufficient in terms of the robustness of the findings. However, since no study has empirically demonstrated this, that may be interrelated and have an effect on confidence. If we assume that there is a correlation between metacognitive ability and bias, then the structure of the metacognitive model would also change. When the correlation between metacognitive ability and bias is explicitly incorporated into the model, the following modification is a possible formula for generating confidence.


(10) $$\begin{array}{c} confidence_{st}∼SkewNormal\left(\mu_{st},\frac{1}{meta_{s}},bias_{s}\right) \end{array}$$

In the above equation, the confidence generation process is represented by a skew normal distribution. The skew normal distribution is a probability distribution that expresses the skewness of the distribution by adding a skewness parameter to the normal distribution, where μ*_*st*_* means the value that the confidence should take, and in this research, it is expressed by the probability of a correct response and a regression model using the *Q* value. Further, by assuming the skewness parameter to be a bias, we can represent a phenomenon in which the confidence is rated biased in one direction. If the bias parameter takes a large value, either positive or negative, then the fringe of the distribution will extend to the right or left, resulting in lower precision.

The main limitation of our study is that we did not examine the validity of experimental manipulation. It is difficult to accurately portray actual metacognitive processes in a cognitive modeling approach. Particularly, whether our model parameter truly reflects metacognitive ability should be explored by future research, as this was not possible during this study. To solve this problem, experimental manipulation is needed (Wilson and Collins, 2019). For instance, a comparison between situations where metacognitive ability is limited (e.g., dual tasks) should prove beneficial. If the metacognitive parameter is altered employing this manipulation, the reflection of metacognition by this parameter would be corroborated. As a best practice, we have to conduct experiment manipulation on metacognitive ability and confirm its responsiveness (Busemeyer and Diederich, 2010; Wilson and Collins, 2019).

Moreover, metacognition may be related to working memory (Fleming and Frith, 2014). Accordingly, a dual-task situation may restrict metacognitive ability. Conversely, the metacognitive evaluation was influenced by reward and punishment conditions (Hacker et al., 2008; Arnold et al., 2016). Similarly, punishment feedback allows us to gauge the accuracy of metacognitive evaluation and reveals other ways to test whether metacognitive training (MCT) produces improvements in metacognitive ability. Notably, Moritz and Woodward (2007) developed MCT for patients with schizophrenia, who were tasked with reasoning under conditions of uncertainty and were promoted to shift their faith and estimate the more correct direction. If we can manipulate the metacognitive ability parameters in our metacognitive model via MCT, the validity of our metacognitive model will be enhanced.

Our contribution to metacognition research lies in developing a novel measure for metacognitive ability. The advantages of our model are that (1) it is less restrictive in its measurement of cognitive performance and confidence, (2) it can be applied to various cognitive tasks encompassing learning and memory, and (3) it is easy to interpret. Lastly, our model has potential applications in a variety of research contexts where measuring metacognitive ability is required.

## Data Availability Statement

The datasets and codes presented in this study can be found in online repositories. The names of the repository and URL can be found here: Open Science Framework (https://osf.io/8kjtu/).

## Ethics Statement

The studies involving human participants were reviewed and approved by the Ethics Committee on Research with Human Participants at School of Human Sciences, Senshu University. The patients/participants provided their written informed consent to participate in this study.

## Author Contributions

KS and YK conceived the study design, planned data analysis, and wrote the manuscript. KS built the model, collected the data, and conducted data analysis. Both authors contributed to the article and approved the submitted version.

## Conflict of Interest

The authors declare that the research was conducted in the absence of any commercial or financial relationships that could be construed as a potential conflict of interest.

## Publisher’s Note

All claims expressed in this article are solely those of the authors and do not necessarily represent those of their affiliated organizations, or those of the publisher, the editors and the reviewers. Any product that may be evaluated in this article, or claim that may be made by its manufacturer, is not guaranteed or endorsed by the publisher.

## Footnotes

1
https://github.com/metacoglab/HMeta-d

## Endnotes

^1^Participants responded to the following items. The order in which the questionnaire and the experiment were administered was counterbalanced.

• Age

• Sex

• Grade

• FLANDERS Handedness Questionnaire

• Metacognitions Questionnaire-30

• Cognitive Failure Questionnaire

^2^Participants responded to the following items before experiment tasks.

• Age

• Sex

• Grade

• Generalized Anxiety Disorder-7

• Patient Health Questionnaire-9
